# Upper helical reconstruction during aesthetic facelift procedure: a case report

**DOI:** 10.1080/23320885.2024.2311729

**Published:** 2024-02-03

**Authors:** Zachary N. Goldberg, David S. Goldberg

**Affiliations:** a Sidney Kimmel Medical College, Thomas Jefferson University, Philadelphia, Pennsylvania, USA; b Department of Otolaryngology – Head and Neck Surgery, Thomas Jefferson University, Philadelphia, Pennsylvania, USA; c Department of Plastic Surgery, Monterey Peninsula Surgery Center, Monterrey, California, USA

**Keywords:** Helix, facelift, pre-auricular flap, random pattern flap, ear reconstruction, trauma

## Abstract

We present a case of a two-stage reconstruction of a traumatic right upper helix deformity using a random pattern layover skin flap in conjunction with an aesthetic facelift procedure. This serves to encourage reconstructive surgeons to be mindful about seeking opportunities to address additional patient concerns when appropriate and safe.

## Introduction

The ear has an intricate anatomical framework that presents a challenge to reconstructive surgeons. Trauma and neoplasm are the most common causes of acquired deformity [1, 2]. Patients that wear eyeglasses can be impacted by defects to the upper third of the helix, specifically the eminence of the posterior triangular fossa, where balancing the arms of glasses is maintained. Occasionally, patients may present with additional aesthetic complaints that provide an opportunity to correct multiple issues in one sequence. This case report describes a two-stage delayed reconstruction of a full-thickness traumatic right upper helix deformity using a pre-auricular, random pattern layover skin flap in conjunction with an aesthetic facelift procedure.

## Case presentation

**Figure 1.**
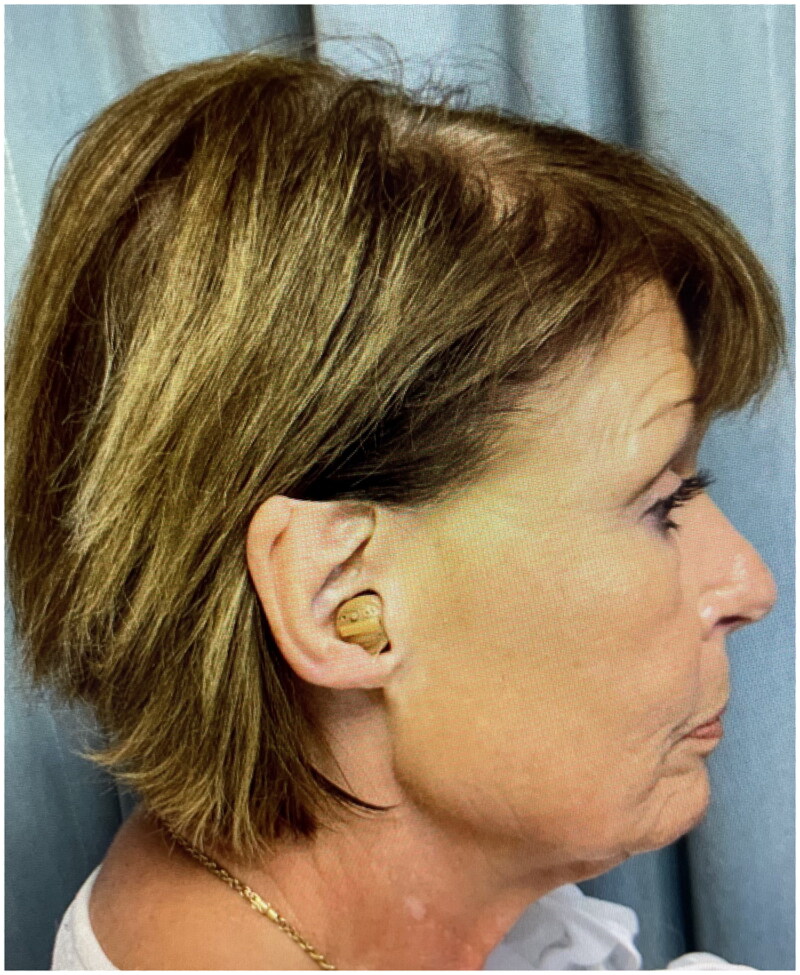
2.5 × 1.5 cm full-thickness defect extending from the right mid-helical crus to the level of Darwin’s tubercle that included the skin and cartilage.

A 68-year-old female presented for concerns related to facial aging, specifically lower facial jowl formation. She also incidentally mentioned previous loss of the right upper middle helix after a traumatic injury when she was in her early 20s. There was a 2.5 × 1.5 cm full-thickness defect from the right mid-helical crus to the level of Darwin’s tubercle that included the skin and cartilage (Figure 1). Additionally, the eminence of the triangular fossa was scarred and attached to the post-auricular scalp skin, preventing the patient from wearing glasses normally. The opportunity for ear reconstruction with retained skin (as a graft or flap) from the facelift procedure and a cartilaginous graft was offered. However, the patient preferred a procedure in which only retained skin would be utilized, and informed consent was obtained.

**Figure 2.**
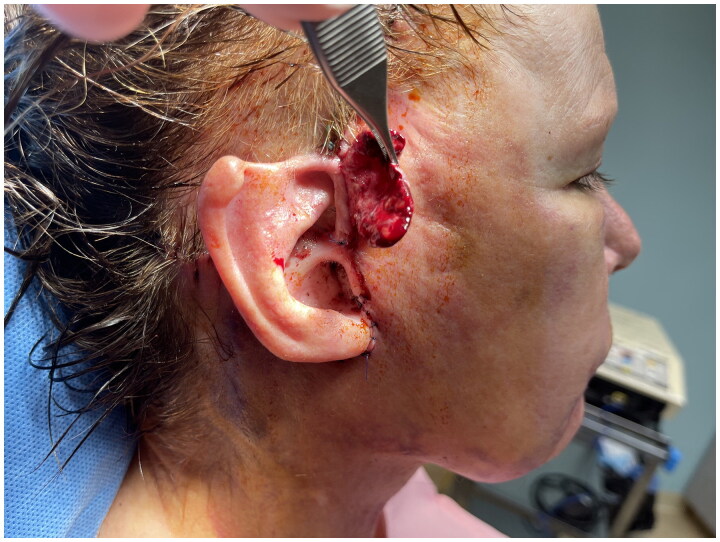
A random pattern flap of retained cheek skin measuring 3.5 × 2.5 cm was elevated and later dressed with antibiotic ointment and gauze to ensure viability and promote maturation for a period of approximately two weeks between stages 1 and 2 of procedure.

In conjunction with the facelift procedure, an incision was made along the upper mid-portion of the right helix to the root of the ear and the scalp. During completion of the facelift procedure, a 3.5 × 2.5 cm local skin flap of retained preauricular right cheek skin was elevated (Figure 2) and dressed with antibiotic ointment and xeroform gauze. To ensure viability and tissue maturation of the large flap, it was re-dressed daily for approximately two weeks.

**Figure 3.**
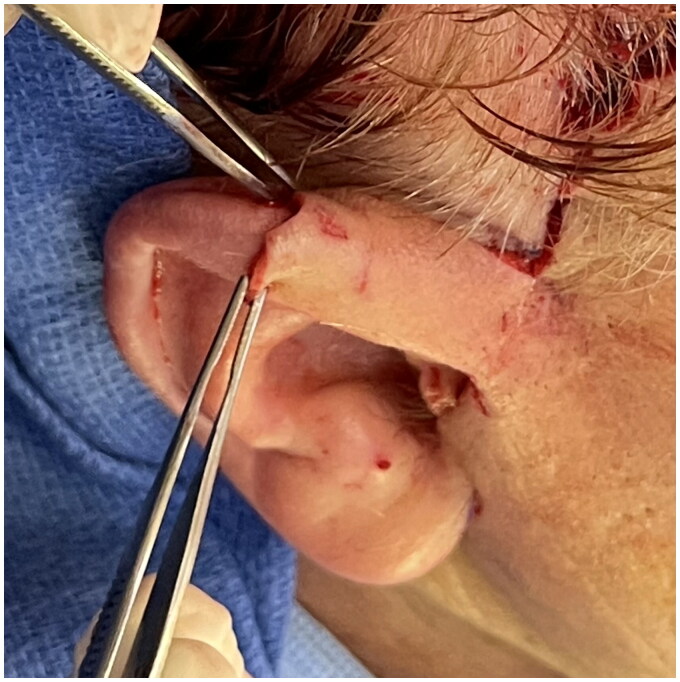
A very similar flap in a different patient (obtained with consent) in which in the inferior portion is rotated backwards toward the upper helix and inset on top of the posterior potion of the existing mid-helix to supplement the eminence of the triangular fossa, and the superior portion is then released and inset on the anterior portion of the helix.

**Figure 4.**
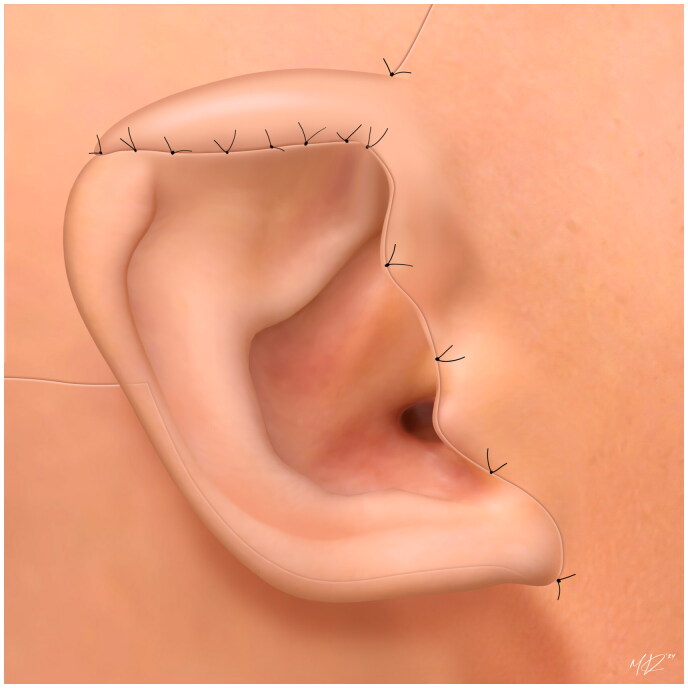
An illustrated diagram of a tubed flap in which in the inferior portion is rotated backwards toward the upper helix and inset on top of the posterior potion of the existing mid-helix to supplement the eminence of the triangular fossa, and the superior portion is then released and inset on the anterior portion of the helix. *Illustrator*: Mario Victor Ramirez Regalado.

After this delay, the patient returned to the operating room again to inset the flap. A 2.5 cm incision was made along the ridge line of the deformed portion of the right helix. The inferior portion of the flap was rotated backwards toward the upper helix and inset on top of the posterior potion of the existing mid-helix to supplement the eminence of the triangular fossa. A tubed flap was created and tacking sutures were placed along the posterior and anterior margins of the existing helix towards the helical root. The superior portion of the flap was then released and inset on the anterior portion of the helix in tubed fashion. A very similar flap in another patient (Figure 3) and an illustrated diagram of this flap is included (Figure 4). The flap was secured with interrupted fine 6-0 non absorbable prolene sutures to recreate the root of the helix at the level of the scalp. Care was taken to avoid tension on all the suture lines, and sutures were removed on post-op day ten.

## Results

**Figure 5.**
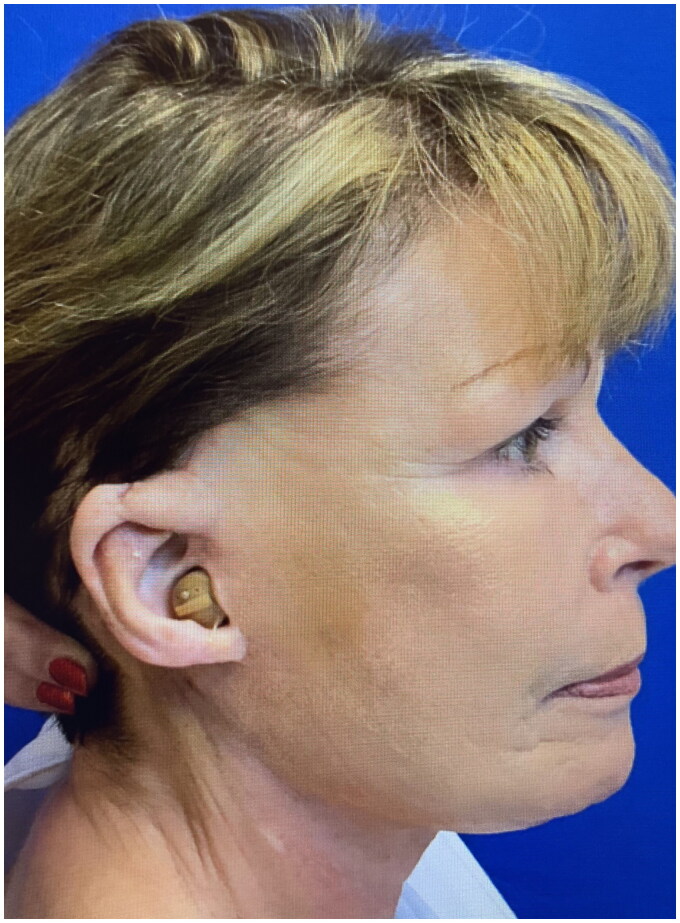
Results of procedure in lateral view two weeks after completion of stage 2 (flap inset).

**Figure 6.**
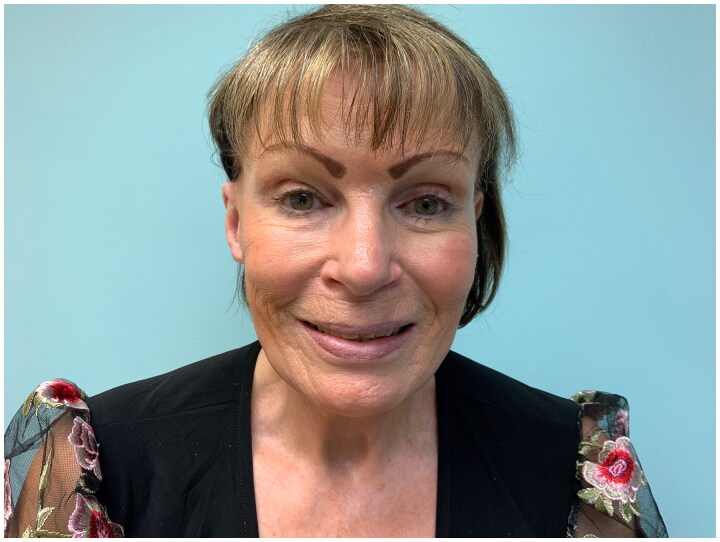
Anterior view of patient 11 months after completion of stage 2 (flap inset) in which the reconstructed right ear is visible and appears symmetrical with the normal contralateral (left) ear helix.

There were no postoperative complications, and the facelift was otherwise uneventful and followed a standard post-operative course. The facial and right helical aesthetic results improved significantly following stage 2 (Figure 5) and at 11 months post-op (Figure 6), and the patient was able to place glasses on the posterior ear sulcus without difficulty. Additional revision procedures were offered, but the patient declined.

## Discussion

This pathology represents a full-thickness, large upper helical defect [1, 3]. Defects of this type require local tissue rearrangement with random or axial flaps [1, 4, 5]. They may require additional composite grafting with cartilage from the antihelix or conchal bowl to repair and stabilize the underlying helical framework [5]. There are various approaches to helical reconstruction using local flaps, including the Antia-Buch flap, Davis flap, Peninsular Conchal Axial Flap, and Pre- or Post-auricular pull-through flap with or without tunneling [1, 3]. Use of a pre- or post-auricular flap has been shown to produce favorable aesthetic and physiologic outcomes in patients with traumatic injuries [2]. Helical reconstruction with these flaps is often performed in multiple stages [1, 3]. The process includes creation of a tubed flap with intact superior and inferior portions, severing of one portion with inset to the defect, and then severing of the other portion with primary closure [1, 3]. Random flaps may be harvested in a delayed manner to improve circulation and promote viability [4].

This procedure was completed in two stages. The first stage involved elevation of the random skin flap with a delay to promote viability, and the second stage involved rotation of the superior and inferior potions of the flap with inset into the defect. A pre-auricular flap was chosen for this reconstructive procedure due to the concurrent desire for an aesthetic facelift procedure. Facelifts require bilateral pre-auricular incisions that extend into the upper scalp skin, which can also be suitable for elevation of tissue for a pre-auricular flap [6]. Given the risk of positional ear deformity as a result of facelift procedure (the ‘pixie ear’ deformity), minimizing excess pre-auricular skin removal is essential for aesthetic success [6]. The pre-auricular tissue used for the random pattern skin flap would have been otherwise discarded in a facelift procedure, but instead was suitable for a reconstructive flap.

The key limitation of this case involves the potential of the ear reconstruction to promote visibility of the facelift due to increased distance from the lateral canthus to temporal hairline. Additionally, while a cartilaginous graft may have improved the stability of the helical rim in conjunction with a local flap, the patient preferred a reconstructive procedure without cartilage grafting. Lack of structural support due to the absence of a graft may lead to cupping and poor cosmetic outcomes in some patients.

## Conclusion

This case report represents a novel use of retained pre-auricular tissue as a delayed random pattern flap to reconstruct the upper helix during an aesthetic facelift procedure. Patients with upper ear deformity also desiring an aesthetic facelift procedure may benefit from surgical resolution of both concerns at the same time. This case serves to encourage reconstructive surgeons to be mindful about seeking opportunities to address additional patient concerns when appropriate and safe.
